# MYD88 L265P mutation in primary central nervous system lymphoma is associated with better survival: A single-center experience

**DOI:** 10.1093/noajnl/vdab090

**Published:** 2021-07-07

**Authors:** Olimpia E Curran, Michael T C Poon, Louise Gilroy, Antonia Torgersen, Colin Smith, Wael Al-Qsous

**Affiliations:** 1 Cellular Pathology, University Hospital of Wales, Cardiff CF14 4XW, UK; 2 Neuropathology Unit, Department of Pathology, Western General Hospital, Edinburgh EH4 2XU, UK; 3 Usher Institute, University of Edinburgh, Edinburgh EH16 4UX, UK; 4 Department of Clinical Neurosciences, Royal Infirmary of Edinburgh, Edinburgh EH16 4SA, UK; 5 Molecular Pathology, Western General Hospital, Edinburgh EH4 2XU, UK; 6 Department of Pathology, Western General Hospital, Edinburgh EH4 2XU, UK

**Keywords:** MYD88 L265P, overall survival, primary central nervous system lymphoma, treatment

## Abstract

**Background:**

The *myeloid differentiation primary response gene* (*MYD88*) mutation in primary central nervous system lymphomas (PCNSL) may be associated with unfavorable prognosis; however, current evidence remains limited. We aimed to characterize PCNSLs by integration of clinicopathological, molecular, treatment, and survival data.

**Methods:**

We retrospectively identified and validated 57 consecutive patients with PCNSLs according to the 2017 WHO classification of lymphoid neoplasms over 13 years. Formalin-fixed paraffin-embedded tumor samples underwent polymerase chain reaction assay to detect *MYD88* mutation. We used Cox regression for survival analysis, including age, treatment, and *MYD88* as covariates. We searched the literature for studies reporting demographics, treatment, *MYD88*, and survival of PCNSL patients and incorporated individual patient data into our analyses.

**Results:**

The median age was 66 years and 56% were women. All 57 patients had PCNSL of non-germinal center cell subtype and the majority (81%) received either single or combined therapies. There were 46 deaths observed over the median follow-up of 10 months. *MYD88* mutation status was available in 41 patients of which 36 (88%) were mutated. There was an association between *MYD88* mutation and better survival in the multivariable model (hazard ratio [HR] 0.277; 95% confidence interval [CI]: 0.09-0.83; *P* = .023) but not in a univariable model. After incorporating additional 18 patients from the literature, this association was reproducible (HR 0.245; 95% CI: 0.09-0.64; *P* = .004).

**Conclusions:**

Adjusting for confounders, *MYD88*-mutant PCNSL appears to show improved survival. While further validation is warranted, detection of *MYD88* mutation will aid the identification of patients who may benefit from novel targeted therapies.

**Key Points**

- *MYD88* mutation in PCNSLs is associated with better survival after adjusting for age at diagnosis and treatment.
- Identification of *MYD88* mutation in PCNSLs could identify patients who may benefit from novel targeted therapies and enhance survival.

**Importance of the Study**

PCNSLs are rare and associated with lower survival than their systemic counterparts. The emergence of new molecular targets in PCNSLs, such as mutations in the *MYD88* gene, offers hope for more effective therapeutics. Few studies have investigated the association between *MYD88* mutation and survival. These studies, however, are limited by inconsistent inclusion of clinical variables and suboptimal analytic approaches, leading to incomplete adjustment for important confounders and model overfitting. Our study integrates treatment, molecular, and survival data for 57 patients diagnosed with PCNSL. We demonstrate that without adequate adjustment for confounders, such as age at diagnosis and treatment, *MYD88* mutation does not affect survival. However, a multivariable survival model including these variables shows *MYD88* mutation to be associated with better survival. While further validation of this association is warranted, our findings suggest that the detection of *MYD88* mutation can identify patients who may benefit from novel targeted therapies and enhance survival.

Malignancies of lymphoid origin in the brain are rare and associated with poor survival.^1^ The most common diagnostic entity is a primary central nervous system lymphoma (PCNSL), defined as diffuse large B-cell lymphoma (DLBCL) arising within the brain, spinal cord, leptomeninges, or eye with no evidence of disease elsewhere.^2,3^ The majority of cases exhibit a non-germinal center cell origin by immunohistochemistry (IHC), corresponding to late germinal center exit B cells with blocked terminal B-cell differentiation.^3,4^ Brain biopsy is considered the gold standard for PCNSL diagnosis.^5^ Treatment options include single therapies, such as chemotherapy or radiotherapy, or a combination of both, or with an autologous stem cell transplantation (ASCT).^5^ Chemotherapy options are typically adapted from systemic regimens, which are compromised by their limited ability to cross the brain-blood barrier.^6^ The emergence of new therapies designed for molecular targets, such as *MYD88* signaling pathway,^7^ may offer additional treatment options for patients affected by these rare tumors.

The *MYD88* L265P mutation is frequent in PCNSLs and has recently been identified as a potential diagnostic marker.^8–12^ The *MYD88* gene codes for a B-cell signaling adaptor protein.^13^ A switch of amino acid leucine to proline at position 265 (L265P) leads to constitutive activation of the nuclear transcription factor kappa B (NF-κB) signaling. This pathway is frequently dysregulated in systemic DLBCLs,^7,10^ and *MYD88* signaling pathway has been evaluated as a potential therapy for DLBCLs. Ibrutinib, a selective inhibitor of Bruton tyrosine kinase (BTK), has also been successfully tried in systemic and CNS DLBCLs with *MYD88* mutation.^14–17^

The prognostic value of *MYD88* L265P mutation in PCNSLs remains inconclusive. Several studies reported no effect^9,18–20^ or unfavorable outcome on overall survival (OS) in PCNSLs.^21,22^ A recent meta-analysis of *MYD88* mutation in DLBCLs from any site has shown no impact on OS.^10^

In this study, we reviewed 57 PCNSLs diagnosed at a single neuropathology center. We retrospectively tested formalin-fixed paraffin-embedded (FFPE) brain tissue for the presence of *MYD88* L265P mutation and assessed its associations with OS taking clinicopathological features and treatment regimens into account. We validated our findings in a larger cohort of PCNSLs identified through a systematic literature search.

## Materials and Methods

### Patients

Histological sections of 57 PCNSLs diagnosed at our center between January 1, 2007, and March 1, 2020, were retrieved from the archives. Clinical characteristics collected from the neuropathological reports included sex (female or male), age (<60, 60-69, or 70+ years), location of lesion (deep or superficial), and tumor extent (unilateral or bilateral). Information about tumor location and extent were confirmed with imaging reports. We searched local clinical databases for information about received treatment regimes, follow-up, and survival. The ethical approval for this study was waived by the Tissue Governance committee of the South East Scotland SAHSC BioResource.

### Histopathology and Immunohistochemistry

Hematoxylin and eosin-stained sections were reviewed in order to confirm diagnosis in accordance with the current 2017 WHO classification of lymphoid neoplasms.^23^ Archived immunohistochemistry-stained sections were reviewed for the expression of B-cell markers, including antibodies against CD20, BCL2, BCL6, MUM1, and CD10. Using the Hans algorithm, cases were further subclassified into germinal B-cell center (GCB) and non-GCB subtypes.^3,24^

### Sample Preparation for MYD88 Mutation Analysis

Genomic DNA was extracted from FFPE tissue blocks and analyzed using a real-time, allele-specific polymerase chain reaction (PCR) analysis. Briefly, DNA was extracted from the FFPE sample using the QIAamp DNA FFPE tissue kit (Qiagen). Real-time allele-specific PCR was performed according to Jiménez et al.^25^ The assay carries a limit of detection of 1% when a minimum of 25 ng DNA is utilized. For all patients in this study, the presence of *MYD88* L265P mutation has been evaluated at diagnosis.

### Data Pooling

A literature search was performed up to May 28, 2020, for published articles in English using PubMed and Embase. The searching details were *MYD88* and primary central nervous system lymphoma. The search was limited to human studies. We excluded patients with systemic DLBCLs, primary vitreoretinal lymphoma, and immunocompromised cases, either HIV- or Epstein Barr virus (EBV) positive as all these entities show distinct clinicopathologic features.^26^We also excluded studies on liquid biopsies. We searched for detailed information about treatment and survival in adult PCNSL patients who were tested for the presence of *MYD88* L265P mutation using molecular techniques. Studies reporting *MYD88* expression using IHC were not included, unless validated with molecular techniques.

### Statistical Analysis

OS was defined as the time from the date of surgery to the date of death or with censoring on the date of last available follow-up. Survival curves were estimated using the Kaplan-Meier method and compared using log-rank test. We used univariable and multivariable Cox regression to assess the effect of *MYD88* mutation and other clinical predictors on OS. The association between *MYD88* mutation and survival was the main effect in the multivariable Cox regression with age and treatment modality as covariates. We chose these covariates because these are the strongest confounders, and we did not include any further putative confounders to avoid overfitting. This approach has several advantages. First, we demonstrated that without taking other variables or confounders into account, *MYD88* mutation is not associated with survival. Second, we illustrated that the effect size for *MYD88* mutation on survival changed when accounting for other covariates. This suggested that there were confounding effects of the included covariates on the association between *MYD88* mutations. These are important findings because previous studies did not take treatment variables into consideration in their survival analyses as shown in our literature review. Without accounting for confounding factors, the association between *MYD88* mutation and survival may be hidden or reversed. We tested proportionality assumption based on Schoenfeld residuals. All survival analyses and graphs were produced with R statistical software (R version 4.0.0) using *tidyverse*, *survminer*, and *survival* packages.

## Results

### Clinical Data

In the period from January 1, 2007, to March 1, 2020, a total of 57 PCNSL patients were diagnosed at our neuropathology center. The median patient age at the time of biopsy was 66 years (range: 31-78 years). Females constituted 56% of patients (female/male ratio 1.3:1). Nineteen (33%) and 38 patients (67%) had a superficial lesion and a deep lesion, respectively. Unilateral involvement was recorded in 42 (74%) patients and bilateral in 15 cases (26%). In all cases, the diagnosis was made on brain tissue biopsy. Following surgery, 25 (44%) patients received a single and 21 (37%) a combination therapy. Chemotherapy combined with radiotherapy, chemotherapy alone, or radiotherapy alone was given to 18 (32%), 17 (30%), and 8 (14%) patients, respectively. Three patients (5%) received autologous stem cell transplant following chemotherapy. Eleven patients (19%) were not fit and did not receive any treatment after the diagnostic biopsy. Details of individual patient treatments are presented in Supplementary Table S1. Details of the distribution of treatments by *MYD88* L265P mutation status testing are presented in Supplementary Table S2. The 1-year, 3-year, and 5-year survival based on Kaplan–Meier estimation were 52% (95% confidence interval [CI]: 40.5-67.6), 22.5% (95% CI: 13.1-38.8), and 7.5% (95% CI: 2.6-21.9), respectively. At the last follow-up, 46 patients had died, and 11 patients were alive. The median follow-up time was 10 months (range: 0-96 months).

### IHC and Molecular Data

All our PCNSLs were DLBCL of non-GCB subtype. *MYC* analysis by fluorescent in-situ hybridization (FISH) was available for 23 cases (15 *MYD88* mutant, 2 *MYD88* wild-type, and 6 *MYD88* mutation status undetermined) none of which showed evidence of an *MYC* rearrangement.

### Data Pooling

Seven studies fulfilled our literature search criteria. Among these studies, a study of Yamada et al.^9^ provided individual patient data on treatment, *MYD88* mutation status, and OS for additional 18 PCNSL cases.

### MYD88 L265P Mutation Analysis

Tissue blocks were not available for 3 (5%) cases. *MYD88* mutation analysis was performed in 54/57 specimens. In 13 (23%) of the cases, the DNA content was too low for reliable testing. There were no major differences in clinical characteristics between the patients with missing *MYD88* data (16/57) and those included in the subsequent survival analyses (41/57) apart from treatment variable (Supplementary Table S3). Overall, good-quality genomic DNA was available for 41 (72%) cases. The *MYD88* c.794T>C substitution status was detected (mutant) in 36 of the 41 (87.8%) patients. There were no significant differences between mutant and wild-type *MYD88* patients (Table 1). There was a tendency for *MYD88* wild-type cases to have a shorter follow-up, but this difference was not statistically significant (2 vs 14 months, *P* = .075). The same analysis performed with additional 18 PCNSL cases from Yamada et al.^9^ showed no significant differences between mutant and wild-type *MYD88* patients. However, the median follow-up was significantly shorter for wild-type patients in comparison to mutated patients (3 vs 15 months, *P* = .045) (Table 2).

**Table 1.** Details of 41 Scottish Primary Central Nervous System Lymphoma Patients With Known MYD88 L265P Mutation Status

Characteristic	Wild-type (*N* = 5)^a^	Mutated (*N* = 36)^a^	*P*-value^b^
Age			>.9
<60 years	1 (20%)	11 (31%)	
60-69 years	2 (40%)	14 (39%)	
70+	2 (40%)	11 (31%)	
KPS			>.9
<70	0 (0%)	2 (8.7%)	
70+	5 (100%)	21 (91%)	
Unknown	0	13	
Sex			.4
Female	4 (80%)	19 (53%)	
Male	1 (20%)	17 (47%)	
Extent			.6
Bilateral	2 (40%)	8 (22%)	
Unilateral	3 (60%)	28 (78%)	
Location			.7
Superficial	1 (20%)	14 (39%)	
Deep	4 (80%)	22 (61%)	
Treatment			.3
None	2 (40%)	6 (17%)	
Single	2 (40%)	12 (33%)	
Combined	1 (20%)	18 (50%)	
Median follow-up (months)	2 (0, 4)	14 (4, 29)	.075

^a^Statistics presented: *n* (%); median (IQR).

^b^Statistical tests performed: Fisher’s exact test; chi-square test of independence; Wilcoxon rank-sum test.

**Table 2.** Details of Pooled Data From 41 Scottish Primary Central Nervous System Lymphoma (PCNSL) Patients and Additional 18 PCNSL Cases From Yamada et al.^9^ With Known MYD88 L265P Mutation Status, *N* = 59

Characteristic	Wild-type (*N* = 6)^a^	Mutated (*N* = 53)^a^	*P*-value^b^
Age			>.9
<60 years	2 (33%)	16 (30%)	
60-69 years	2 (33%)	22 (42%)	
70+	2 (33%)	15 (28%)	
KPS			.3
<70	0 (0%)	9 (22%)	
70+	6 (100%)	31 (78%)	
Unknown	0	13	
Sex			.7
Female	4 (67%)	26 (49%)	
Male	2 (33%)	27 (51%)	
Extent			.2
Bilateral	3 (50%)	12 (23%)	
Unilateral	3 (50%)	41 (77%)	
Location			.3
Superficial	1 (17%)	25 (47%)	
Deep	5 (83%)	28 (53%)	
Treatment			.3
None	2 (33%)	6 (11%)	
Single	2 (33%)	16 (30%)	
Combined	2 (33%)	31 (58%)	
Median follow-up (months)	3 (0, 8)	15 (6, 30)	.045

^a^Statistics presented: *n* (%); median (IQR).

^b^Statistical tests performed: Fisher’s exact test, chi-square test of independence, and Wilcoxon rank-sum test.

### Survival Analysis

**Figure 1.**
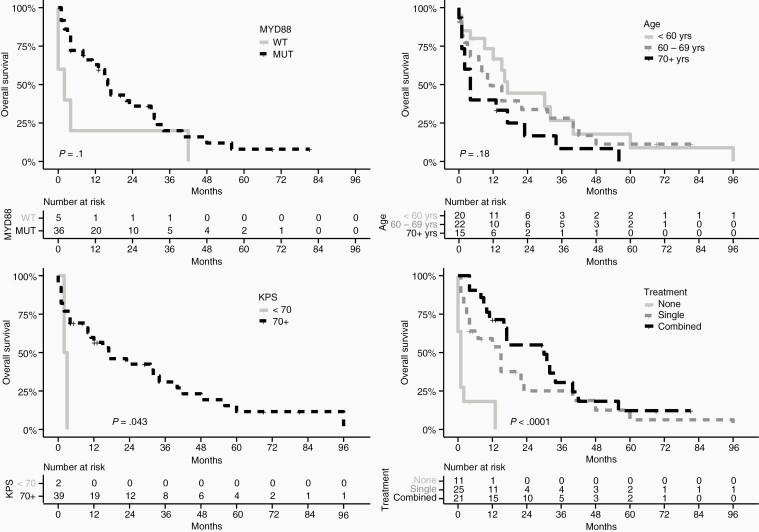
Kaplan-Meier estimation of the overall survival for 57 Scottish primary central nervous system lymphoma patients. The overall survival was defined as the time from the date of surgery to the date of death or with censoring on the date of last available follow-up.

Survival function estimated by Kaplan-Meier method was stratified by *MYD88*, sex, age, treatment, KPS, tumor location, and tumor extent. *MYD88*, age, treatment, and KPS, which are shown in Figure 1. Treatment was associated with better OS, and as expected, treated patients had a significantly increased OS in comparison to untreated patients. The estimated median OS by Kaplan-Meier method for single and combined treatment regimens were 15 (95% CI: 4-40) and 30 (95% CI: 16-56) months, respectively. Median OS for untreated patients was 1 month. Although KPS below 70 showed a significant unfavorable association with OS, there were only 2 cases with KPS below 70 in the Scottish cohort, which should be interpreted with caution. The pooled analysis revealed similar findings for age and treatment (Supplementary Figure S1). KPS showed no survival advantage in the pooled cohort. There was, however, a significant advantage in survival for *MYD88-*mutated PCNSL cases, but the numbers were low (only 6 wild-type *MYD88* patients).

**Figure 2.**
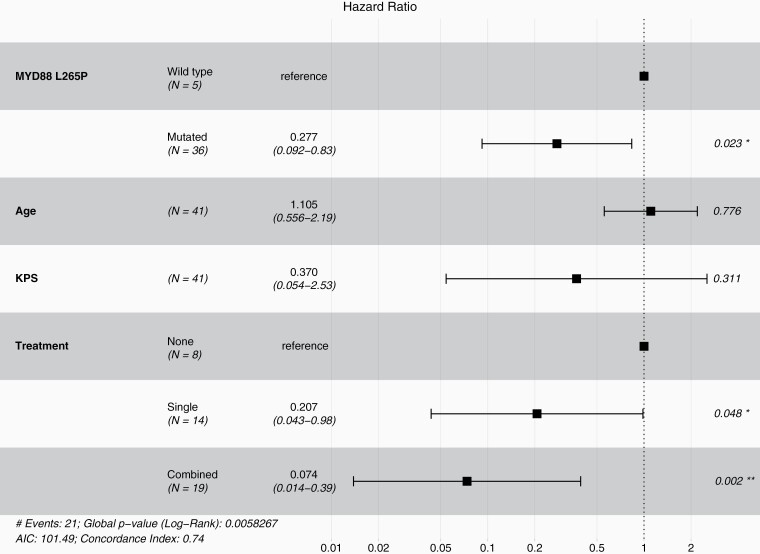
Forest plot for Cox proportional hazards model of multivariate analysis for 41 Scottish primary central nervous system lymphomas with known MYD88 L265P mutation status and treatment showing hazard ratios, 95% confidence intervals, and *P*-values.

**Figure 3.**
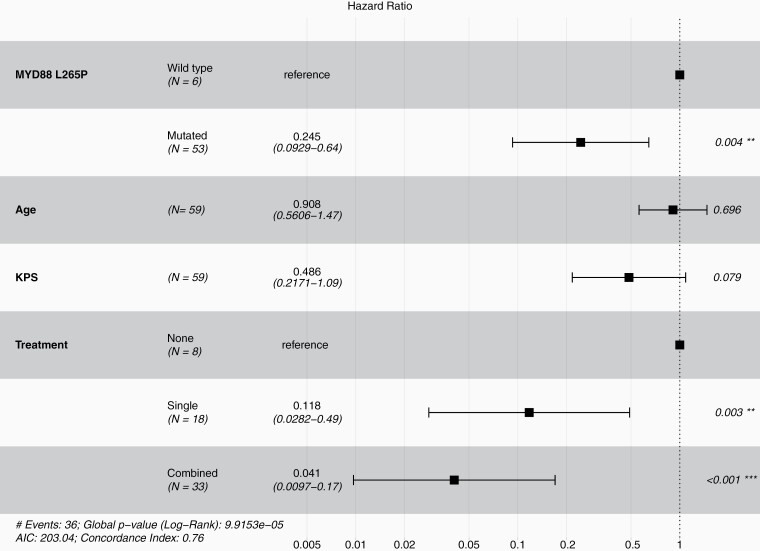
Forest plot for Cox proportional hazards model of multivariate analysis from pooled data of 59 primary central nervous system lymphomas with known MYD88 L265P mutation status and treatment showing hazard ratios, 95% confidence intervals, and *P*-values.

Multivariate analysis of the Scottish cohort (*n* = 41) revealed two significant findings (Figure 2 and Supplementary Table S4). Firstly, it was confirmed that single (*n* = 14) (HR 0.207, 95% CI: 0.043-0.98, *P* = .048) and combined (*n* = 19) (HR 0.074, 95% CI: 0.014-0.39, *P* < .001) treatments were associated with survival advantage to no treatment. Secondly, mutant *MYD88* (*n* = 36) was associated with a significant survival advantage relative to wild-type *MYD88* (*n* = 5) (HR 0.277, 95% CI: 0.09-0.83, *P* = .023). These findings were further validated in pooled multivariate analysis cohort (*n* = 59) (HR 0.245, 95% CI: 0.093-0.64, *P* = .004) (Figure 3 and Table 3).

**Table 3.** Multivariable Cox Regression Survival Analysis From Pooled Data of 59 Primary Central Nervous System Lymphoma Patients With Known MYD88 Mutation Status and Treatment Information From Scottish Cohort (*N* = 41) and Yamada et al.^9^ (*N* = 18)

Characteristic	HR	95% CI	*P*-value
MYD88			
Wild type	—	—	
Mutated	0.24	0.09, 0.64	**.004**
Age	0.91	0.56, 1.47	.7
KPS	0.49	0.22, 1.09	.079
Treatment			
None	—	—	
Single	0.12	0.03, 0.49	**.003**
Combined	0.04	0.01, 0.17	**<.001**

HR, hazard ratio; CI, confidence interval.

A significant level was set to *P* < .05.

## Discussion

This retrospective, large single-center study describes 57 PCNSLs with emphasis on the significance of the *MYD88* L265P mutation on OS in immunocompetent patients who received different treatment regimens. We show that *MYD88* mutation in PCNSLs appears to be associated with a survival advantage. Considering the increasing importance of molecular markers in prognostication and treatment strategy, our results highlight emerging clinicopathological and molecular factors for this rare patient group with PCNSL.

Consistent with previous reports,^9,13,18,27–32^ we found a high frequency of the *MYD88* L265P mutation in PCNSLs. A recent meta-analysis by Lee et al.^10^ reported the prevalence of this mutation in 59.8% (95% CI: 42.2-75.2) of CNS cases. The higher frequency of 88% reported in our study may reflect all cases belonging to non-GCB subtype of PCNSL. This subtype is known to be more common in PCNSLs.^31^ Moreover, non-GCB lymphoid malignancies from other sites have higher rates of this particular mutation.^10,13^ Non-GCB subtype is characterized by constitutive activation of NF-κB (nuclear factor kappa-light-chain enhancer of activated B cells) pathway,^33^ while *MYD88* mutation is an oncogenic driver of NF-κB pathway.^30^ It remains uncertain, however, as to why CNS lymphomas have an increased incidence of dysregulated NF-κB signaling at a site, the CNS, which is also considered to be immune-privileged.^30,34^ This may explain why other mutations, in addition to the *MYD88* L265P, are necessary to coexist in order to have a prognostic impact in these rare lymphomas.^8,29,34^

In contrast to the previous studies, our findings suggest that the presence of mutant *MYD88* is associated with better survival in PCNSL patients. The prognostic value of *MYD88* mutation in systemic DLBCLs has been debated for some time with studies providing data for both sides of the argument.^10^ For PCNSLs several studies reported no effect on OS^9,11,19,20^ with only 2 studies reporting an unfavorable outcome.^21,22^ There are, nevertheless, important methodological differences between ours and the previous reports making direct comparisons between the studies difficult. Discrepancies may be due to different analytic approaches. Association between molecular markers and survival is often confounded by clinical variables. Moreover, only 3 of the 7 identified studies^9,18–22,35^ performed multivariate analyses; 2 reporting adverse impact on OS^21,22^ and one showing no difference.^18^ Furthermore, the discrepancies may reflect differences in the biological properties of the PCNSL subtypes. Our patients with PCNSL were exclusively non-GCB subtype and all but one^21^ included studies did not report on DLBCL subtypes. Previous studies reported limited treatment variables,^18^ with some recruited only patients treated with chemotherapy.^21,22^ Selection bias is likely to contribute to the heterogeneous findings on the association between *MYD88* mutation and survival. Lastly, there may also be genetic differences between the different patient cohorts with the three studies originating from Japan.

Our data suggest that among patients with treated PCNSL, a combined-modality therapy may be better at prolonging survival. A recent study of PCNSLs reported that a significantly prolonged survival could be achieved with a combined-modality therapy, although, unlike in our study, autologous stem cell therapy was excluded.^36^ The later half of this data collection period includes evolving treatment paradigms, largely through optimization of dose-intensive immunochemotherapy. The inclusion of novel agents, which are highly effective at crossing the blood-brain barrier, such as thiotepa in the MATRix regimen, has been demonstrated to be associated with improved clinical outcomes in a disease previously considered incurable^37^ leading it to be considered the new standard of care. Thiotepa-based therapies, however, are difficult to implement in countries with limited resources.^36^ Combined-modality therapies may prolong survival but tend to lead to significant neurotoxicity. Newer treatments designed to reflect molecular tumor background may be more effective if they are target-specific. In fact, therapeutic agents targeting *MYD88* mutations have already been tested in clinical settings. Ibrutinib, a BTK inhibitor, inhibits NF-κB signaling pathway and has been used for treatments of systemic non-GCB DLBCLs^14^ and PCNSLs.^16,38^ An 83% partial response rate was reported in a recent clinical trial of ibrutinib in PCNSL.^16^ Our findings suggest that identification of the *MYD88* L265P mutation in PCNSLs may help evaluate the benefits associated with the use of targeted therapy in these patients.

### Strengths and Limitations

This study included the largest cohort of patients with PCNSL with clinical, molecular, and treatment variables available. We were able to investigate the effect of *MYD88* mutation on survival accounting for major confounders. Results from the pooled cohort from the literature also supported our observation. However, investigating a rare type of tumor inevitably has issues of power. We only included age, KPS, and treatment in the multivariate analysis because these are the most important confounders to *MYD88* mutation. This also avoided overfitting of the data. While it would be of interest to investigate other prognostic factors, we were unable to do so. The existing literature on PCNSL is heterogeneous and pooling of individual patient data was challenging. We only included patients with treatment information since treatment is a strong predictor of survival. Though this did not allow a larger cohort to be established, our pooled analyses included only patients with adequate clinical features to be informative. An important limitation of our study is the small number of PCNSL patients with the wild-type *MYD88.* Patients with wild-type *MYD88* had less favorable clinical factors such as fewer treatments and older age. While the multivariable model adjusted for these factors, the small sample size contributed to the imprecision of our effect size estimate. The rarity of wild-type *MYD88* and changing clinical management of PCNSL call for a concerted collaborative effort for investigating the role of *MYD88* mutation in the survival of patients with PCNSL.

Consistent with previous reports, this study shows that *MYD88* L265P mutation is common in PCNSLs. Our multivariable analysis incorporating clinical variables shows that *MYD88* mutation is associated with favorable survival in patients with PCNSL, suggesting the confounding effects of clinical factors. Studies with a larger cohort with contemporary clinical management can clarify the prognostic value of *MYD88* mutation and evaluate the potential benefit from therapeutic agents targeting *MYD88* mutations.

## Supplementary Material

vdab090_suppl_Supplementary_Materials

## Notes

**Authorship Statement**. O.E.C.—study design, data acquisition, analysis and interpretation, and manuscript preparation; M.T.C.P.—data analysis and interpretation and manuscript preparation; L.G.—MYD88 analysis; A.T. and C.S.—pathology reporting; W.A.-Q.—concept and study design, data acquisition, and pathology reporting. All authors reviewed the final manuscript.
