# Supplementary material for: MYD88 L265P mutation in primary central nervous system lymphoma is associated with better survival: A single-center experience
Source: Neurooncol Adv. 2021 Jul 7;3(1):vdab090. doi: 10.1093/noajnl/vdab090 (PMC8349182; doi:10.1093/noajnl/vdab090)
Supplement: vdab090_suppl_Supplementary_Materials [file vdab090_suppl_supplementary_materials.docx]

**Title: MYD88 L265P mutation in primary central nervous system lymphoma is associated with better survival: A single centre experience.**

Olimpia E. Curran^1^, Michael T. C. Poon^2,3^, Louise Gilroy^4^, Antonia Torgersen^1^, Colin Smith^1^, Wael Al-Qsous^5^.

SUPPLEMENTARY TABLES (4)

SUPPLEMENTARY FIGURES (1)

SUPPLEMENTARY TABLES

Table S1. Details of treatment regimens for 57 PCNSL patients from Scottish cohort.

| Regimen | Management group | Details of treatment | No. of cases |
| --- | --- | --- | --- |
| Single | CMT | HD-MTX | 5 |
|  |  | DeAngelis | 5 |
|  |  | Matrix | 7 |
|  | RT | WBRT | 4 |
|  |  | WBRT + RTt | 1 |
|  |  | WBRT palliative | 3 |
| Combined | CMT & RT | DeAngelis + WBRT | 6 |
|  |  | DeAngelis + WBRT + TM | 3 |
|  |  | HD-MTX + RTt | 1 |
|  |  | HD-MTX + WBRT | 3 |
|  |  | Matrix + WBRT | 5 |
|  | CMT & ASCT | Matrix + ASCT | 3 |
| None | None | None | 11 |

ASCT, autologous stem cell transplant;

CMT, chemotherapy;

CMT & RT, chemotherapy and radiotherapy;

CMT & ASCT, chemotherapy and autologous stem cell transplant;

HD-MTX, high-dose methotrexate;

RT, radiotherapy;

RTt, radiotherapy targeted;

TM, temozolomide;

WBRT, whole brain radiotherapy.

Table S2. Distribution of 57 Scottish PCNSL cases stratified by MYD88 L265P mutation status testing and treatment management.

| MYD88 | Cases (%) | CMT | RT |  | CMT&RT | CMT&ASCT | None |
| --- | --- | --- | --- | --- | --- | --- | --- |
|  |  | Single | | | Combined | |  |
| Mutated | 36 (63%) | 9 | 3 |  | 17 | 1 | 6 |
| Wild type | 5 (9%) | 0 | 2 |  | 0 | 1 | 2 |
| Failed | 13 (23%) | 7 | 2 |  | 1 | 1 | 2 |
| Not tested | 3 (5%) | 1 | 1 |  |  |  | 1 |
| Total | 57 | 17 | 8 |  | 18 | 3 | 11 |

CMT, chemotherapy;

CMT & RT, chemotherapy and radiotherapy;

CMT & ASCT, chemotherapy and autologous stem cell transplant;

RT, radiotherapy.

Table S3. Comparison of missing (without known MYD88 L265P mutation status) and non-missing (with known MYD88 L265P mutation status) patients for Scottish cohort, N=57.

| Characteristic | Missing, N = 16*^1^* | Non-missing, N = 41*^1^* | p-value*^2^* |
| --- | --- | --- | --- |
| **Age** |  |  | 0.2 |
| <60 years | 8 (50%) | 12 (29%) |  |
| 60-69 years | 6 (38%) | 16 (39%) |  |
| 70+ | 2 (12%) | 13 (32%) |  |
| **KPS** |  |  | >0.9 |
| <70 | 0 (0%) | 2 (7.1%) |  |
| 70+ | 13 (100%) | 26 (93%) |  |
| Unknown | 3 | 13 |  |
| **Sex** |  |  | >0.9 |
| Female | 9 (56%) | 23 (56%) |  |
| Male | 7 (44%) | 18 (44%) |  |
| **Extent** |  |  | 0.7 |
| Bilateral | 5 (31%) | 10 (24%) |  |
| Unilateral | 11 (69%) | 31 (76%) |  |
| **Location** |  |  | 0.6 |
| Superficial | 4 (25%) | 15 (37%) |  |
| Deep | 12 (75%) | 26 (63%) |  |
| **Treatment** |  |  | 0.031 |
| None | 3 (19%) | 8 (20%) |  |
| Single | 11 (69%) | 14 (34%) |  |
| Combined | 2 (12%) | 19 (46%) |  |
| *^1^*Statistics presented: n (%)  *^2^*Statistical tests performed: Fisher's exact test; chi-square test of independence | | | |

Table S4. Hazard ratios from Cox multivariate analysis from Scottish PCNSLs patients with known MYD88 L265P mutation status and known treatment regimens (N=41).

| **Characteristic** | **HR***^1^* | **95% CI***^1^* | **p-value** |
| --- | --- | --- | --- |
| **MYD88** |  |  |  |
| Wild type | — | — |  |
| Mutated | 0.28 | 0.09, 0.83 | **0.023** |
| **Age** | 1.10 | 0.56, 2.19 | 0.8 |
| **KPS** | 0.37 | 0.05, 2.53 | 0.3 |
| **Treatment** |  |  |  |
| None | — | — |  |
| Single | 0.21 | 0.04, 0.98 | **0.048** |
| Combined | 0.07 | 0.01, 0.39 | **0.002** |
| *^1^*HR = Hazard Ratio, CI = Confidence Interval | | | |

SUPPLEMENTARY FIGURE

Figure S1. Kaplan-Meier estimation of the overall survival based on data available for 75 PCNSL patients from pooled analysis (Yamada et al., (N=18) and the Scottish study, (N=57)).
